# Intrusion Experiments to Measure Territory Size: Development of the Method, Tests through Simulations, and Application in the Frog *Allobates femoralis*

**DOI:** 10.1371/journal.pone.0025844

**Published:** 2011-10-14

**Authors:** Max Ringler, Eva Ringler, Daniela Magaña Mendoza, Walter Hödl

**Affiliations:** Department of Evolutionary Biology, University of Vienna, Wien, Austria; University of Western Ontario, Canada

## Abstract

Territoriality is a widespread behaviour in animals and its analysis is crucial in several areas of behavioural, ecological and evolutionary research. Commonly, territory size is assessed through territory mapping and the application of simple area estimators such as minimum convex polygons. In the present study we demonstrate that territory size can be determined adequately with an active approach through intrusion experiments, a technique that is commonly used in behavioural research in other contexts. Tests with simulated data indicate that a minimum of twelve trials needs to be performed to establish reliable orders of relative territory size. To estimate absolute territory size, detailed hull techniques are most appropriate when analyzing point patterns of intrusion experiments, while the local convex hull estimator enables the construction of internal utilization distributions based on such point patterns. Additionally we suggest a ‘stretch the centre’ approach to emphasize the actual process of intrusion experiments in the construction of internal utilization distributions. To demonstrate the utility of the method, we apply all findings from the simulations to data from fieldwork with the model species *Allobates femoralis*, a territorial aromobatid frog from the lowland rainforest of French Guiana.

## Introduction

The territory of an animal is usually defined as an area of intense and often exclusive use, which is announced and delimited by visual, acoustic, chemical and/or electric cues. In most cases a territory is also defended by physical aggression against conspecifics, but sometimes also heterospecific intruders of either one or both sexes [1]. This opposes the concept of a territory to that of a home range, which is defined as the entire area used by an individual in its regular activities [2]. The functional inequality of a territory and a home range has further implications regarding which data to use to adequately describe the one or the other. For example in birds, it was shown that singing locations alone do not provide an accurate estimate of space use [3] as the animals use and defend much wider areas than those delimited by the sites that are preferred for singing.

Usually a territory contains one to several resources an animal needs to sustain its life, such as shelter or feeding resources, and/or to allow or support its reproduction, such as display sites, nesting sites or sites for egg or larval deposition [4]. It can be derived logically that, all other things being equal, territories of larger size are more likely to contain any of the resources mentioned, or to contain any one of these resources in higher quantities or qualities. In turn, higher resource abundance can allow for smaller territories, especially in the light of trade-offs between costs and benefits of large territories [5] (but see also [6]).

The ability of an individual to defend a territory of larger size has been shown to be a reliable indicator of an individual's quality and/or social status within a population, to be evaluated by conspecific competitors of equal sex and potential mating partners of the opposite sex [7], [8]. Likewise, territory size has been shown to be linked to parameters of individual fitness like number of mates [9] or reproductive success [10] (but see [11] for contrasting findings in *Allobates femoralis*). While ‘true’ absolute territory size estimates might be of special interest for management and conservation purposes [12], individual-focussed correlational studies, for example on reproductive behaviour and sexual selection, at least need reliable estimators for relative territory size among a group of individuals [13], [14]. Thus suitable estimators for these purposes have to produce concise and reliable rank orders of territory sizes, while absolute territory size is often only of secondary interest.

The most widely used approach to assess territory extension is through ‘territory mapping’ [15], the observation of focal individuals and their marking and delimiting behaviour, as well as their interactions with other individuals. This yields points to define the centres of activity as well as points of interaction and delimiting behaviour at the periphery of the area an individual is defending [16], [17]. Subsequently, these point patterns can be evaluated with a variety of area estimators, the most common ones (cf. [18], with a focus on home range studies) being minimum convex polygons (MCP) [19] and parametric (*sensu* [20]) kernel methods [21]. However, this purely observational approach to study territory size is susceptible to the observer's chance and ability to detect a sufficient number of peripheral locations for all individuals under study [22], [23] to get individually unbiased estimates of territory extension. Additionally, the observations have to be situated in space and time in a way to allow for concise estimates of territory size without exceeding biases in either dimension [24]–[26].

**Figure 1:**
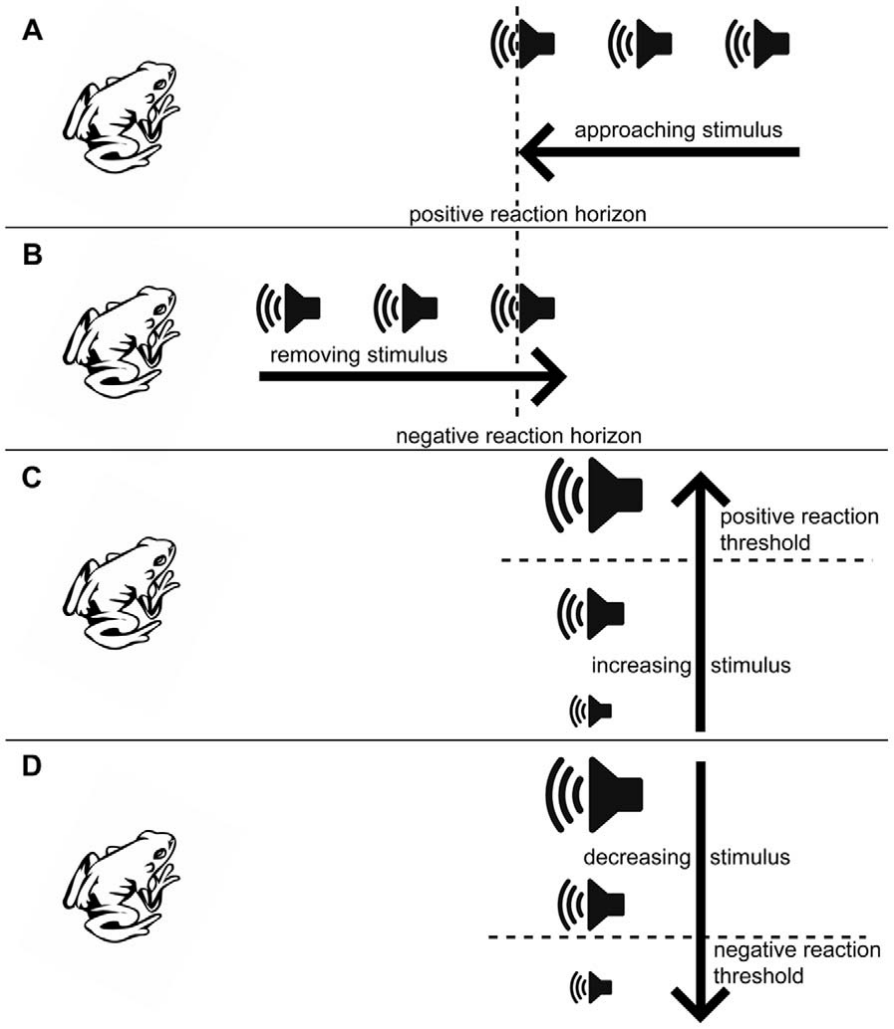
Setup of intrusion experiments. There are four general setups for intrusion experiments with A) approaching or B) removing stimuli at fixed intensities, or C) increasing or D) decreasing stimuli at fixed distances.

Especially in situations where territorial individuals display territory ownership from central sites but defend wider areas against intruders [27], an active, systematic assessment of territory size can be preferable when obtaining territory sizes for a larger number of individuals. For this purpose the respective territorial response in a species needs to be elicited actively by the researcher to observe territoriality ‘in action’. This can be achieved through the performance of intrusion experiments, where adequate cues of fixed intensity are displayed towards focal individuals, or other operational entities such as a breeding colony, at decreasing distances (Fig. 1A), to find out about the reaction horizon, the maximum distance at which a territorial response can be elicited. This method can also be reversed, so that an adequate cue of a fixed intensity is presented at a distance where a response is reliable (e.g. in 95% of all trials) and subsequently the cue is removed until no response is observable (Fig. 1B). Alternatively, the intensity of the cue can be increased at a fixed distance until a response is noticed (Fig. 1C), or conversely, decreased at a fixed distance, until no response can be elicited (Fig. 1D). The data gained in a fixed cue-location setup then needs to be calibrated to allow the calculation of a reaction horizon (for various applications of the method cf. [28]–[35]). The actual approach taken depends on the type of cue that is used to elicit territorial behaviour, the type of territorial response, and possible and sometimes long term, reactions like stress, hiding behaviour, or territory desertion of individuals of a given species elicited by such experiments. Furthermore, the time available in terms of territory stability as well as experimenter time, the number of experimenters available, and the characteristics of the environment in which the territories are found will influence the decision for or against one or the other approach.

Surprisingly, despite the regular appearance of intrusion experiments in the behavioural literature, to the best of our knowledge no consensus method for the evaluation of the point patterns typically produced in these experiments has been reported. In general, a reliable territory estimator should be able to yield estimates with low bias and high precision, independently of the actual behaviour of a certain study species [36]. Additionally, point pattern shape and sample size sample size should not severely affect the estimator [37], [38]. With detailed evaluations of existent estimators lacking and no new estimators that were developed explicitly for the use with point patterns from intrusion experiments, studies generally will fall back on MCPs as the simplest of all area estimators. However, this approach has two major drawbacks. MCPs, like all approaches that simply connect points, do not produce an internal utilization distribution as is obtained by kernel methods, and they are rather sensible to the number of points in an analyzed pattern. Typically MCPs reach an asymptote well beyond the maximal number of intrusion experiments that reasonably can be performed on a single individual [38], [39]. This can be alleviated by the use of detailed-hull techniques, where a certain set of restriction rules defines which points are connected to delimit a point pattern [40]. Thus, the assessment of absolute territory size is also exacerbated by the general problem of information theory of how to fit shapes to a set of points in a plane [41], [42].

In this study we describe the method of intrusion experiments to assess territory size and find the most suitable area estimators for the point patterns produced in these experiments. This is achieved by evaluating such point patterns from simulations and a dataset from fieldwork with the territorial dendrobatoid frog *Allobates femoralis*. A focus is given on free plug-ins in the ArcView©/ArcGIS© (ESRI) software environment, which are widespread among field biologists, to ensure the practical applicability of the method.

## Materials and Methods

### Simulated data

**Figure 2:**
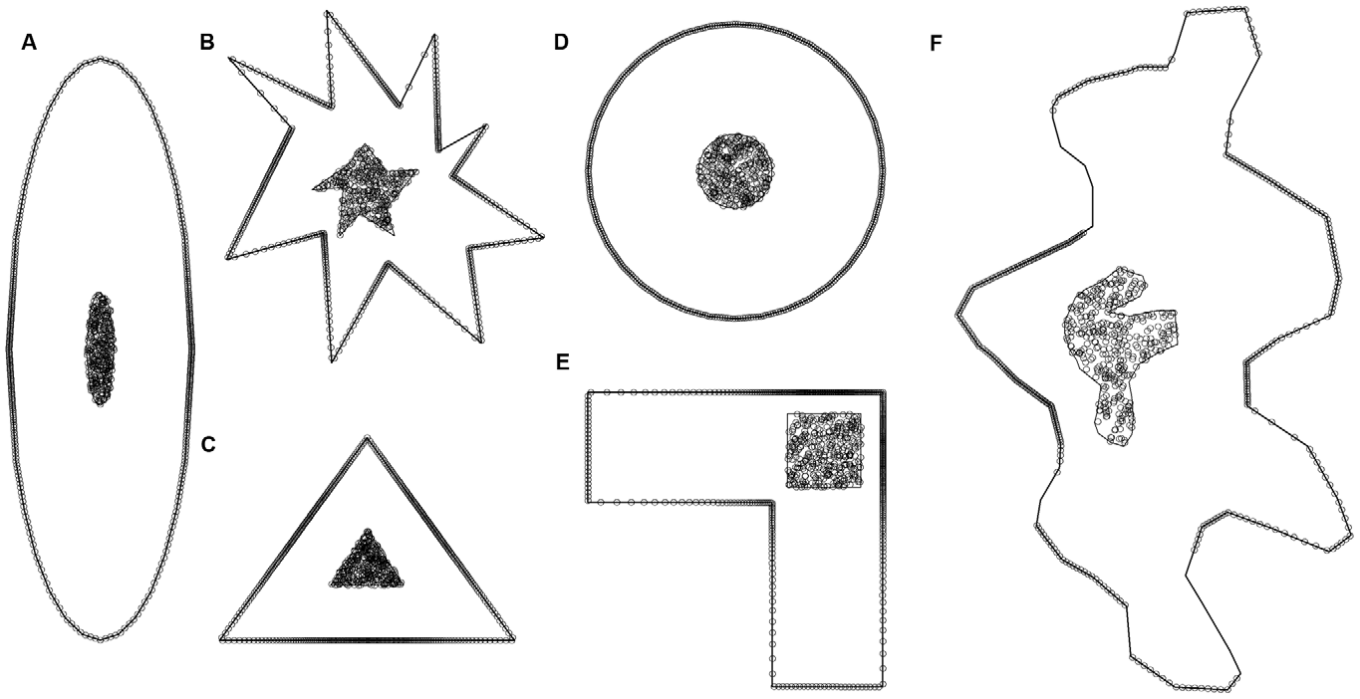
Shapes of the virtual territories. A) ellipse, B) star, C) triangle, D) angle, E) circle, F) irregular. The territories comprise points from simulated intrusion experiments consisting of 360 randomly placed starting points in the central areas and an equal number of trial endpoints that were placed on the border of each territory, equiangularly in relation to the centroid points of the central areas.

**Figure 3:**
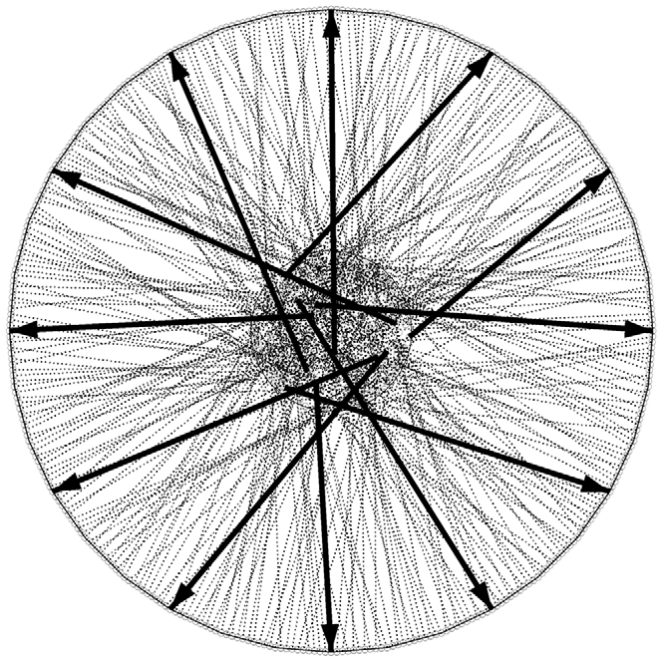
Circular virtual territory. Circular virtual territory with all possible 360 simulated intrusion trials (dotted lines) and the indication of an equiangular subset of twelve trials (bold arrows).

To investigate the performance of different territory estimators in the analysis of point patterns as produced from intrusion experiments, and to decide on the actual number of trials to be performed in field experiments, we constructed six virtual territories (Fig. 2). The territories were drawn in ArcMap (ESRI) to represent a range of shapes from strictly convex to highly concave, and to span a range of sizes (‘true’ absolute territory sizes in arbitrary units: ellipse: 243.58, star: 130.09, triangle: 88, circle: 200.04, angle: 155.63, irregular: 395.11). Each territory consisted of a central area, representing assumed display and resting sites, and an outer region, representing a wider defended area. This mimicked the typical central-place territorial behaviour of many species, where potential intruders are detected and intercepted well before reaching an individual's area of concentrated use. The central areas of each territory were populated with 360 random points, using ‘Hawth’s Tools' for ArcMap [43], while along the edge of the outer regions 360 points were placed at 1°-intervals in relation to the centroid of the central area. Random pairs of one central and one edge point were grouped to represent simulated intrusion trials consisting of an initial position in the central area and a final position at the edge of the outer area (Fig. 3). For each of the six territories we described all 810 possible equiangular trial-subsets within the 360 directions with numbers of trials that are integer divisors of 360 (i.e. 2, 3, 4, 5, 6, 8, 9, 10, 12, 15, 18, 20, 24, 30, 36, 40, 45, 60, 72, 90, 120, 180, 360). The rationale behind using equiangular trial sets, compared to trials in random directions, was to ‘span up’ the entire extension of a territory with a minimum number of trials to minimize time effort for experiments and disruptions and stress caused on the focal animals by excessive testing.

### Frog data

Our research on *Allobates femoralis* in the CNRS field station “Saut Pararé” in the Nature Reserve "Les Nouragues", French Guiana was approved by the scientific board of the station (http://www.nouragues.cnrs.fr/F-conseil.html). No formal permits or approval ID″s were issued by this board, as our experiments did not involve killing or harming animals. All experiments were conducted according to French and EU law and followed the ASAB guidelines for the treatment of animals in behavioural research and teaching [44].

### Study species

*Allobates femoralis* is a small, diurnal frog in the family Aromobatidae that inhabits the leaf litter throughout Amazonia and the Guiana shield [45]. The species forms disjunct local populations in lowland rainforests that are not exposed to regular inundations (i.e. “terra firme” forests). Males announce their multi-purpose territories [46] by prolonged and intense calling during the reproductive season [47] and defend these territories against calling intruders by vigorous physical attacks [48]. Obviously the territory plays a vital role in the elaborate courtship that precludes mating and can last over several hours [49], [50]. Females show a high degree of site fidelity but do not defend any territories [51]. In a recent study [11], territory occupancy was shown to be the main determinant of male reproductive success, while territory size, measured as in previous studies by mapping encounter locations [46], [51], did not influence the quantitative performance of actually reproducing males.

### Study site and mapping

Field data were gathered by two experimenters (MR, ER) during the rainy season from February 28^th^ until March 16^th^, 2009 in a population of *A. femoralis* near the field station ‘Saut Pararé' (4°02′ N, 52°41′ W; WGS84) in French Guiana, France in the course of a long term research project on reproductive behaviour and space use in this species. All spatial data were recorded in ArcPad 6.0 (ESRI) on PocketPCs (iPaq HX4700, Hewlett-Packard) using a detailed background map of all living trees (dbh >10 cm), fallen trees and larger branches, and other structures on the forest floor that were used by frogs or constituted landmarks, to map the locations of the frogs.

### Playback Trials

**Figure 4:**
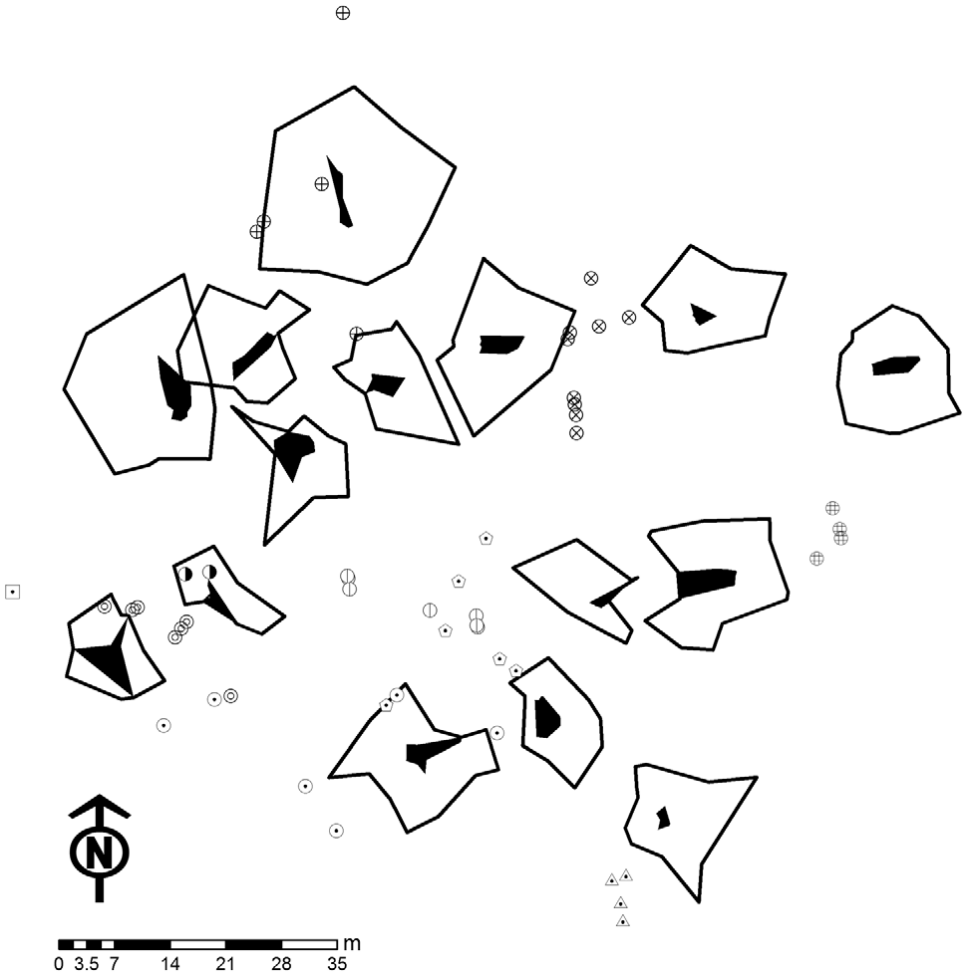
Spatial setup of 15 focal *Allobates femoralis* males during intrusion experiments. Black areas shows the detailed hull of the calling positions, outer bold lines shows the detailed hull of the endpoints of playback trials. Symbols indicate the encounter locations of ten other males that were found in the area during the intrusion experiments.

To assess the area of defended territories we conducted playback experiments on 15 neighbouring males in our study population (Fig. 4). Our approach took advantage of the stereotypic phonotactic behaviour of *A. femoralis* males [48], [52] and used synthetic advertisement calls from a previous study on phonotactic approach patterns in this species [53]. The calls were presented to the focal animals from WAV-files via a portable audio player (Maxfield G-Flash 512) and battery powered portable loudspeakers (Sony SRS-M30; frequency range: 250–20.000 Hz). All individuals were tested in twelve runs in a semi-random order. The number of twelve trials for each frog was chosen based on the results of the analysis of the simulated dataset (cf. results and discussion of the simulated data). The twelve playback trials per individual were conducted in a semi-random order towards every 30° (0° ≙ north). We picked a random direction for the first trial for each individual, the second trial was performed in the complementary direction (+180°) and the third and fourth trial per individual were performed in random order to the right (+90°) and to the left (+270°). For the fifth trial a random direction was picked among the remaining directions and trials six to eight were performed in a similar manner to trials one to four. Finally, trials nine to twelve were performed accordingly within the remaining four directions. This protocol enabled us to uniformly ‘span up’ the territories of all individuals, thus avoiding unwanted temporal or spatial concentrations of data points for any individual over the trial period.

Before each trial, the initial position where the frog was spotted, usually when calling, was entered into the digital map. Then we continuously played bouts of ten synthetic calls (‘standard call’ *sensu* [53]) towards the focal male from the selected trial direction and from a distance of three meters (±30 cm, depending on vegetation structure). We kept the speaker 20 cm above ground level and adjusted to produce a SPL of more than 56 dB at the position of the focal individual to elicit phonotactic behaviour [54]. As soon as the frog started its phonotactic approach, the speaker was carefully withdrawn in the respective direction to maintain an equal distance (3 m ±50 cm) between the focal individual and the speaker throughout the trial. Trials ended when a frog did not further approach the speaker during three consecutive bouts of the artificial call (∼30 sec). The final location of the frog at the end of each trial was entered in the digital map, then the frog was caught, a picture of the ventral pattern was taken for identification, and finally the frog was released at its initial position. No frog was tested more than two times per day, and always with more than 4 hours between consecutive trials. When a frog did not respond in a playback trial, it was immediately tested in another direction. When the second trial resulted in a phonotactic approach, only the latter trial was recorded and a trial in the previous direction repeated later. When the frog did not respond again, other trials in these directions were performed on later days. When the frog approached the speaker in these later trials, only the successful approaches were scored, otherwise only the initial locations were retained with no corresponding final locations.

### Estimators of territory size and utilization distribution

#### Simulated data

Thirteen different territory estimators were calculated for all 810 equiangular simulated trial sets per virtual territory in the GIS programs ArcMap© 9.3.1 and ArcView 3.3© (ESRI) with commonly used plug-in extensions. Minimum convex polygons (MCP) [19] and detailed hulls (DH) were calculated with the plug-in ‘XTools Pro 4.2.0’ [55] in ArcGIS. The detailed hull method as implemented in XTools Pro selects the points for hull construction similar to the point selection algorithm of [56]. Normal bivariate fixed kernel estimators [21], [57] with least-squares-cross-validation (LSCV) and with an ad-hoc method (ADHOC) to find the smoothing parameter *h* were calculated with the ‘Animal Movement Extension’ (AM) [58] in ArcView. LSCV and a reference value (HREF) for the smoothing parameter were used for similar kernel calculation with the plug-in ‘ABODE’ [59] in ArcGIS, and LSCV, biased cross-validation (BCV) and HREF were used for fixed (f) and adaptive (a) kernel calculations with the plug-in ‘Home Range Tools’ (HRT) [60] in ArcGIS. Local convex hull (LoCoH) nonparametric kernels [61] were calculated as *k*-LoCoHs with the LoCoH-extension [62] in ArcView. The optimal value for the tuning parameter *k* for each territory was selected by applying the ‘minimum spurious hole covering’ (MSHC) rule [20] where *k* is selected manually to avoid any biologically, geographically or topologically unjustified holes or cutaways in the 100%-LoCoH of an individual.

To assess the performance of all estimators, we compared the final area estimates, based on 360 simulated trials per territory, with the known ‘true’ sizes of these territories and evaluated the asymptotic and rank-order behaviour of the estimators with increasing sample size. All polygon estimators, including LoCoH, were evaluated at their full extension, while all parametric kernel estimators were evaluated at the 95% isoclines, the most commonly used extension in home range studies [18]. The minimal number of equiangular intrusion trials that generally has to be performed to reach concise conclusions about relative territory size was evaluated by examining the asymptotic behaviour and rank-order of averaged area accumulation curves of the DH estimator for each shape. This estimator was chosen based on the previously described strong dependence of polygon estimators on the number of points used, where detailed hull methods better fit concave shapes compared to MCPs [40], [42], [63]–[65].

To compare the performance of the different estimators on random, bounded point patterns, we also calculated all estimators, besides the LoCoH estimator, for only the points from the central area with the points at the edge of the outer region omitted.

#### Frog data

We took a stepwise approach in the analysis of the frog data. First, we assessed the extent of the central areas of the territories, resulting from the initial locations of a frog (≙ ‘calling territory’), as well as of the defended outer areas, resulting from the final locations of a frog in the playback trials (≙ ‘playback territory’), using the DH method as it was the best performing estimator with the simulated data. In a second step we derived internal utilization distributions (UD) for the frogs’ territories, based on the entirety of central and peripheral (playback) positions. We used the LoCoH estimator, which was the best performing UD estimator with the simulated data, to emphasize UD calculation based on the entire set of points for a given individual. Additionally we developed a manifest ‘stretch-the-centre’ (STC) method to shape a parametric kernel of the calling positions to the outline of the playback territory, conceptually similar to ‘elastic disc’ models for central-place home ranges [66].

**Figure 5:**
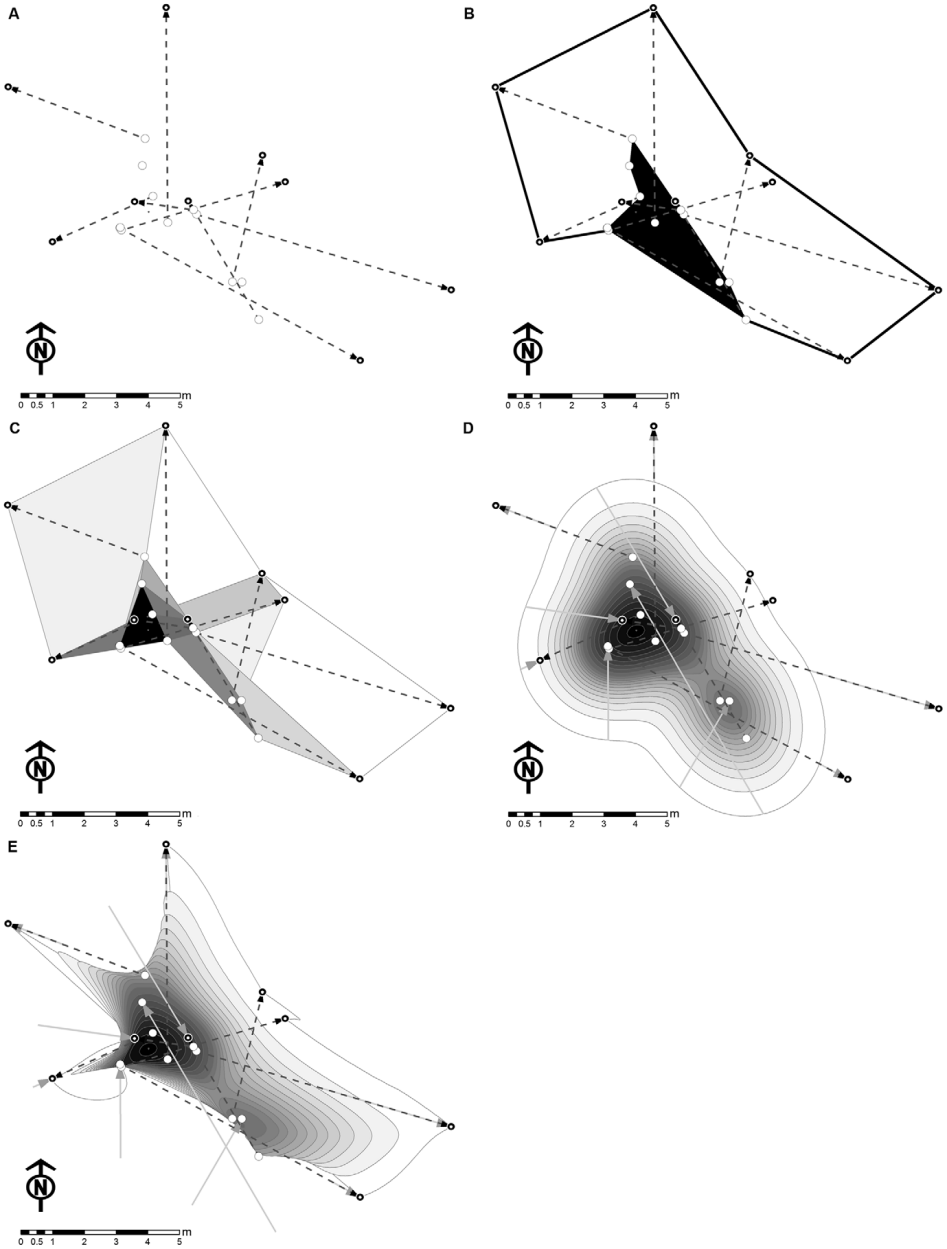
Intrusion trials and territory estimators for focal individual #08. White circles show calling positions, black open circles represent final locations of playback trials, dashed lines show trial vectors and light grey lines indicate correction vectors for STC, areas between kernel isoclines and of n%-LoCoHs (99%, 95-5% (5%-steps), 1%) in incremental shades of grey; A) initial calling positions and final trial positions with corresponding trial vectors, B) DH estimations for calling (black area) and playback territories (bold outline), C) LoCoH estimator with MSHC-adjusted *k*, D) kernel from HRT-HREF based on all calling positions, E) STC transformed kernel.

For STC only the initial positions of the intrusion experiments are used for kernel calculation, as they generally are arranged in a way where parametric kernels were shown to perform well (i.e. bounded random distributions) [57], [67]. Kernels derived from these points are then expanded and reshaped to fit the outer locations from the intrusion trials, which emphasizes the process (i.e. moving animals) that led to the underlying point patterns. As the necessary spatial adjustment operations are not accessible to automation in ArcGIS, we did not integrate this method in our tests with simulated data, due to the excessive manual manipulation required for this approach. Based on the evaluation of the different kernel estimators with the simulated central points (cf. results for simulated data), we calculated HRT-HREF(f) with all observed calling positions for each individual. We then used the ‘rubbersheet’ [68] function for spatial adjustment in ArcMap to reshape each central kernel to the edge of the corresponding defended area by stretching and jolting. For this purpose we linked the 99%-isocline of the central kernel along the axis of the twelve trial vectors (calling position → attracted position) with the corresponding end points of each trial as correction links and set all calling positions as identity links (cf. ArcGIS manual [69]). When an individual showed no response in a given direction, calling position and attracted position were taken to be identical, thus the transformation vector was set along the trial axis, pointing from the 99% isoclines towards the calling position. With all correction links set, we performed the spatial adjustment operation on all isoclines of the central kernel (Figs. 5D, 5E). Similar to other studies that used utilisation distributions from parametric kernels we subsequently analyzed the STC kernels at their 95% isoclines.

#### Statistical Analysis

All descriptive statistics were performed in SYSTAT 12© and SigmaPlot© 11.0. Normality of data was checked with the Shapiro-Wilk test as implemented in SYSTAT. Spearman rank-order correlations were calculated in SigmaPlot. The significance level was set at p<0.05 in all cases.

## Results

### Simulated data

**Figure 6:**
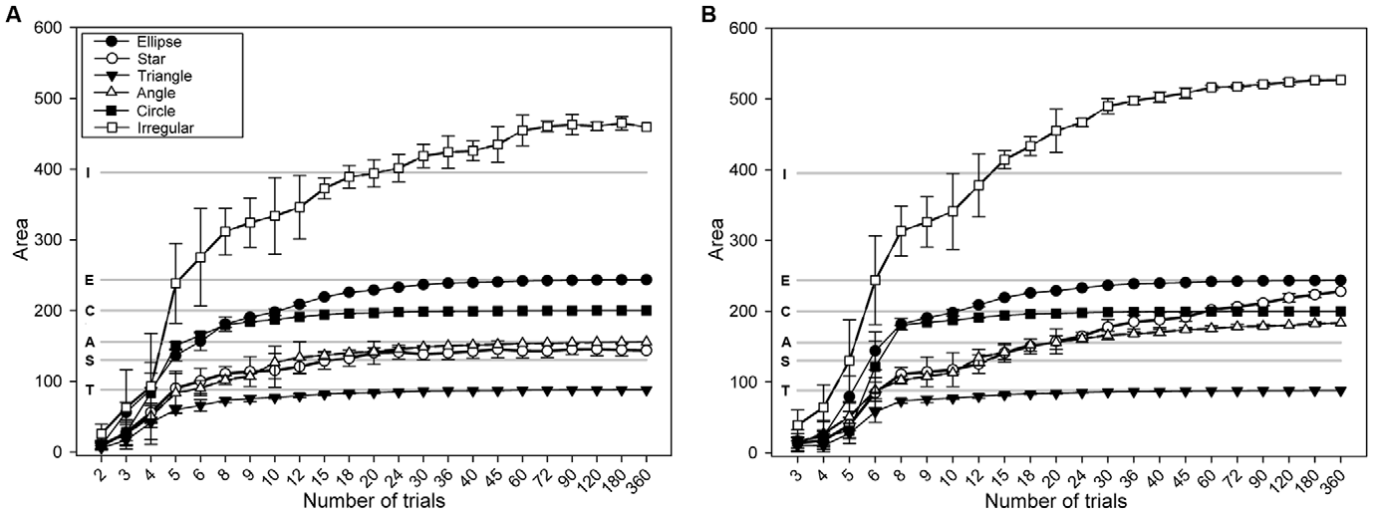
Estimator performance for equiangular trial-subsets in virtual territories. Average values for equiangular subsets; horizontal grey lines indicate ‘true’ absolute territory sizes; error bars indicate standard deviations; area in arbitrary units; A) Detailed hull estimator, B) *k*-LoCoH estimator with MSHC-adjusted *k*.

Both polygon estimators as well as the LoCoH estimator, but none of the parametric kernel estimators, showed stable and asymptotic behaviour with an increasing number of trials in the equiangular trial-subsets over all shapes (Figs. 6A, 6B, S1). When evaluated at the maximum number of trials (ie. 360 trials), the DH estimator reached the ‘true’ absolute territory size most closely for all but the irregular shape, where ABODE-HREF performed slightly better. For strictly convex shapes (i.e. ellipse, triangle, circle) MCP, DH and LoCoH estimators produced identical percentages while with concave shapes (i.e. star, angle, and irregular) the DH estimator performed much better (Table 1). The DH estimator produced a stable rank order for all subsets with ten and more trials, with variation decreasing with an increasing number of trials (Fig. 6A). Absolute territory size of the DH estimator did not increase more than 2% stepwise with an increasing number of equiangular trials from 20 trials upwards for all but the ‘irregular’ territory (Table 2). The rank order of the 100%-LoCoHs with MSHC-optimized *k* remained stable for all convex and the ‘angle’ and ‘irregular’ territories with twelve and more trials, while the estimator for the ‘star’ territory increased continually and changed its rank twice beyond twelve trials (Fig. 6B).

**Table 1:** Performance of estimators based on 360 equiangular intrusion trials for all virtual territories.

Shape	TRUE (units^2^)	MCP	DH	LoCoH	AM -LSCV	AM-ADHOC	ABODE-LSCV	ABODE-HREF
**e**	243.58	**100%**	**100%**	***100%***	35%	37%	56%	103%
**s**	130.09	182%	**110%**	175%	84%	85%	***110%***	140%
**t**	88.00	**100%**	**100%**	***100%***	68%	69%	80%	114%
**a**	155.64	132%	**100%**	118%	67%	68%	76%	*99%*
**c**	200.04	**100%**	**100%**	***100%***	127%	128%	66%	95%
**i**	395.11	138%	116%	133%	71%	71%	83%	***111%***

Shape	TRUE (units^2^)	HRT-LSCV(f)	HRT-BCV(f)	HRT-HREF(f)	HRT-LSCV(a)	HRT-BCV(a)	HRT-HREF(a)
**e**	243.58	17%	125%	125%	21%	167%	167%
**s**	130.09	55%	171%	171%	69%	207%	207%
**t**	88.00	34%	155%	155%	41%	193%	193%
**a**	155.64	28%	147%	140%	33%	187%	181%
**c**	200.04	25%	148%	148%	28%	187%	187%
**i**	395.11	29%	159%	159%	35%	196%	196%

TRUE gives the known size of a virtual territory in units^2^, all other columns give percentages of this area as estimated by the different methods; best performing estimators for a given shape in **bold**, best performing estimators with internal utilization distribution in *italic*; **e**llipse, **s**tar, **t**riangle, **a**ngle, **c**ircle, **i**rregular.

**Table 2:** Relative change in territory size with increasing sample size, calculated with the DH estimator for all virtual territories.

	Number of equiangular trials										
Shape	3	4	5	6	8	9	10	12	15	18	20
**e**	498%	58%	54%	15%	16%	5%	4%	6%	5%	***3%***	1%
**s**	224%	102%	61%	11%	10%	2%	1%	4%	7%	3%	***6%***
**t**	190%	149%	43%	10%	11%	3%	2%	3%	***3%***	2%	1%
**a**	155%	102%	64%	8%	13%	5%	18%	***5%***	2%	2%	1%
**c**	149%	201%	80%	10%	9%	***3%***	2%	2%	2%	1%	0%
**i**	143%	47%	157%	16%	13%	4%	3%	4%	8%	4%	1%

Shape	24	30	36	40	45	60	72	90	120	180	360
**e**	2%	2%	1%	0%	0%	1%	0%	0%	0%	0%	0%
**s**	1%	-2%	1%	2%	2%	-1%	0%	2%	0%	-1%	-1%
**t**	1%	1%	1%	0%	0%	0%	0%	0%	0%	0%	0%
**a**	2%	2%	1%	1%	1%	1%	0%	0%	0%	0%	0%
**c**	1%	0%	0%	0%	0%	0%	0%	0%	0%	0%	0%
**i**	2%	4%	1%	0%	2%	***5%***	1%	1%	0%	1%	-1%

The last step-to-step change >2% in ***bold-Italic*** **; e**llipse, **s**tar, **t**riangle, **a**ngle, **c**ircle, **i**rregular.

**Figure 7:**
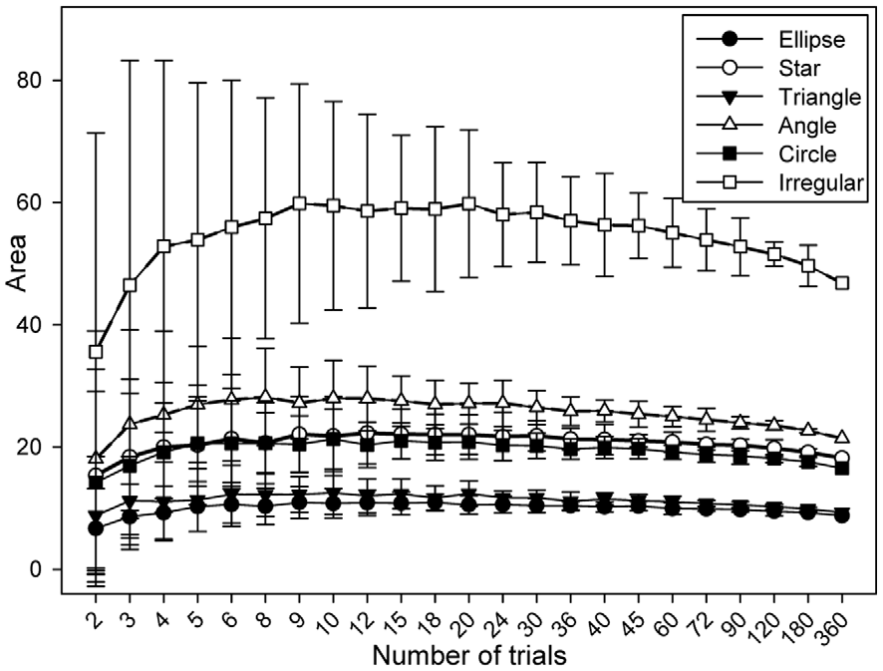
Performance of the HRT-HREF(f) estimator for the central points of the virtual territories. Average values for equiangular subsets; error bars indicate standard deviations; area in arbitrary units.

For the central point distributions the only parametric kernel estimators with a reliable, non-erratic behaviour (Fig. S2) and producing an invariant rank order of territory sizes at a sample size of twelve and more locations were the fixed and adaptive HRT-HREF estimators with the former performing slightly better in terms of stability (Fig. 7). Accordingly we used the fixed HRT-HREF estimator subsequently in our ‘stretch-the-centre’ approach on the frog data.

### Frog data

**Figure 8:**
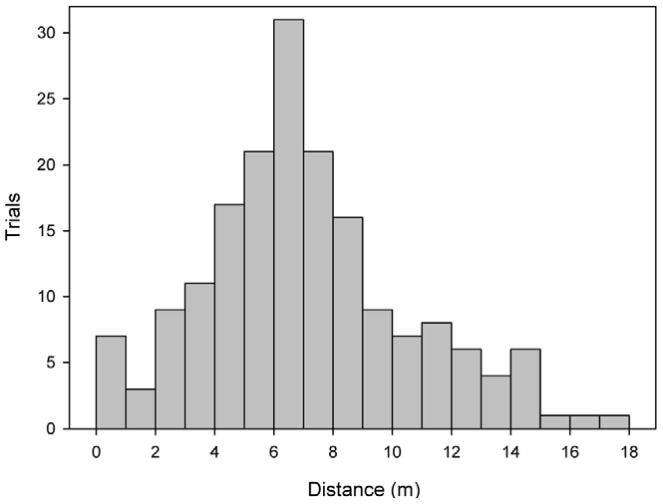
Distance in playback trials. Histogram of the distances that were covered by 15 male *A. femoralis* in playback trials when they were presented with artificial calls to elicit territorial behavior as a reaction to an alleged intruder; bin width  = 1 m.

In 179 playback trials, 15 *Allobates femoralis* males approached the loudspeaker over a mean ± SD distance of 7.07±3.5 m (Fig. 8). In all trials where the males approached the loudspeaker, they unambiguously ended their phonotactic approach at a certain point for at least 30 seconds, thus suggesting that they had reached the border of their defended area. Due to our experimentation protocol we missed one final playback location for two frogs, and two and three final playback locations, respectively for two other frogs. This corresponds to directions into which these frogs apparently did not claim any territory possession. For one frog we could only perform eleven intrusion experiments due to time constraints, but still included the data in the final analyses. Ten other male *A. femoralis* that were encountered in the area during the trials (Fig. 4) were either found outside their territories during tadpole transport, did not show territorial behaviour, or left the trial area after one or two trials, probably because they had not yet established a territory at the time of the first trial and refused to settle after their encounter with the artificial caller. Two males in the centre of the study area were involved in prolonged territory disputes and did not show reliable phonotactic reactions at the onset of the experiments, so we excluded them from the study. None of the frogs ceased territory occupancy during the trials or showed any other evidence of enhanced stress. All frogs started to call again within 10 minutes after being released on their original calling sites.

**Figure 9:**
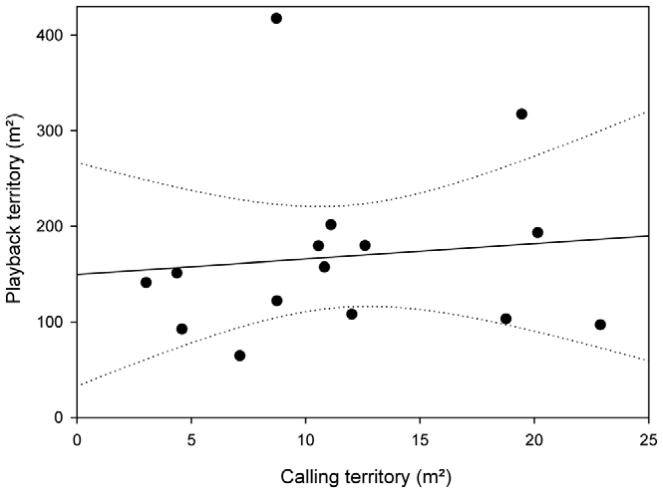
Scatter plot of the extensions of calling territories vs. playback territories. Relation of the area spanned by the initial positions (‘calling territory’) to the area spanned by the final positions (‘playback territory’) in intrusion experiments with 15 male *A. femoralis* as delimited by the DH estimator; linear regression and 95% confidence intervals as continuous and dotted line, respectively.

The DH estimations of the area of the calling territories (range: 3.02 – 22.89 m^2^; median  = 10.82 m^2^,) and playback territories (range: 64.62 – 417.63 m^2^; median  = 151.13 m^2^) varied considerably. All playback territories were larger than their accompanying calling territories by a median factor of 14.54 (range: 4.24 – 47.90). Calling and playback territory extension were not significantly correlated (Spearman ρ = 0.024, *p* = 0.457; Fig. 9). The 100% LoCoH (range: 65.06 – 424.7 m^2^; median  = 164.36) and the 95% STC kernels (range 30.06 – 250.03 m^2^; median  = 94.02 m^2^) showed similar variation among the individuals. The rank order of territory sizes, based on calling and playback points, remained the same for five individuals over all estimators, while for seven individuals the rank varied by one position, for two individuals the rank varied by up to two positions, and for one individual the rank varied for up to three positions among the different estimators (Fig. S3). There was a strong significant correlation between the extension of LoCoH and STC %-isoclines (Spearman ρ = 0.923, *p*<0.001). As it is intrinsically impossible to calculate a 100%-isocline for parametric kernels, the 99% isoclines of the STC estimator were used as an approximation in this correlation analysis. The median %-isocline where the extension of the calling territory (DH) was reached was 50% (range: 30% – 70%) for the LoCoH estimator and 40% (range: 15% – 65%) for the STC estimator. However, due to differential shapes, complete overlap on average was only reached at the 80% (median, range: 50% – 100%) isocline for the LoCoH estimator and the 90% (median, range: 75% – 99%) isoclines for the STC estimator.

## Discussion

### Simulated data

The analysis of point patterns from the simulated intrusion trials in virtual territories showed that central-place territories can be adequately evaluated by at least twelve equiangular intrusion trials. Based on this minimum number of trials, the detailed hull estimator produces reliable rank orders of territory size for further correlational analyses. To estimate absolute territory size however, the number of trials that is necessary for the area accumulation curve to reach an asymptote and thus to reflect the ‘true’ territory size, may exceed the number of trials that reasonably can be performed on individual animals. An animal’s limited tolerance to the stress caused by repeated disturbance and conflict situations with alleged intruders during excessive testing could result in severely altered behaviour, including site abandonment, which would prohibit carrying out the desired number of trials. On the other hand, when activities that should take place in the time frame of the intrusion trials (e.g. reproductive behaviour such as advertisement, mate choice, courtship or mating) subsequently shall be related to territoriality and territory size, the individuals under study have to be given ample time for these activities besides defending their territories against alleged intruders. These factors force a trade-off decision between the accuracy of the territory estimates and the number of trials that can be sustained by the focal individuals. In this context the intended number of trials per individual has to be carefully chosen, and based on our simulated dataset we suggest twelve trials to be a reasonable starting point for future studies.

**Figure 10:**
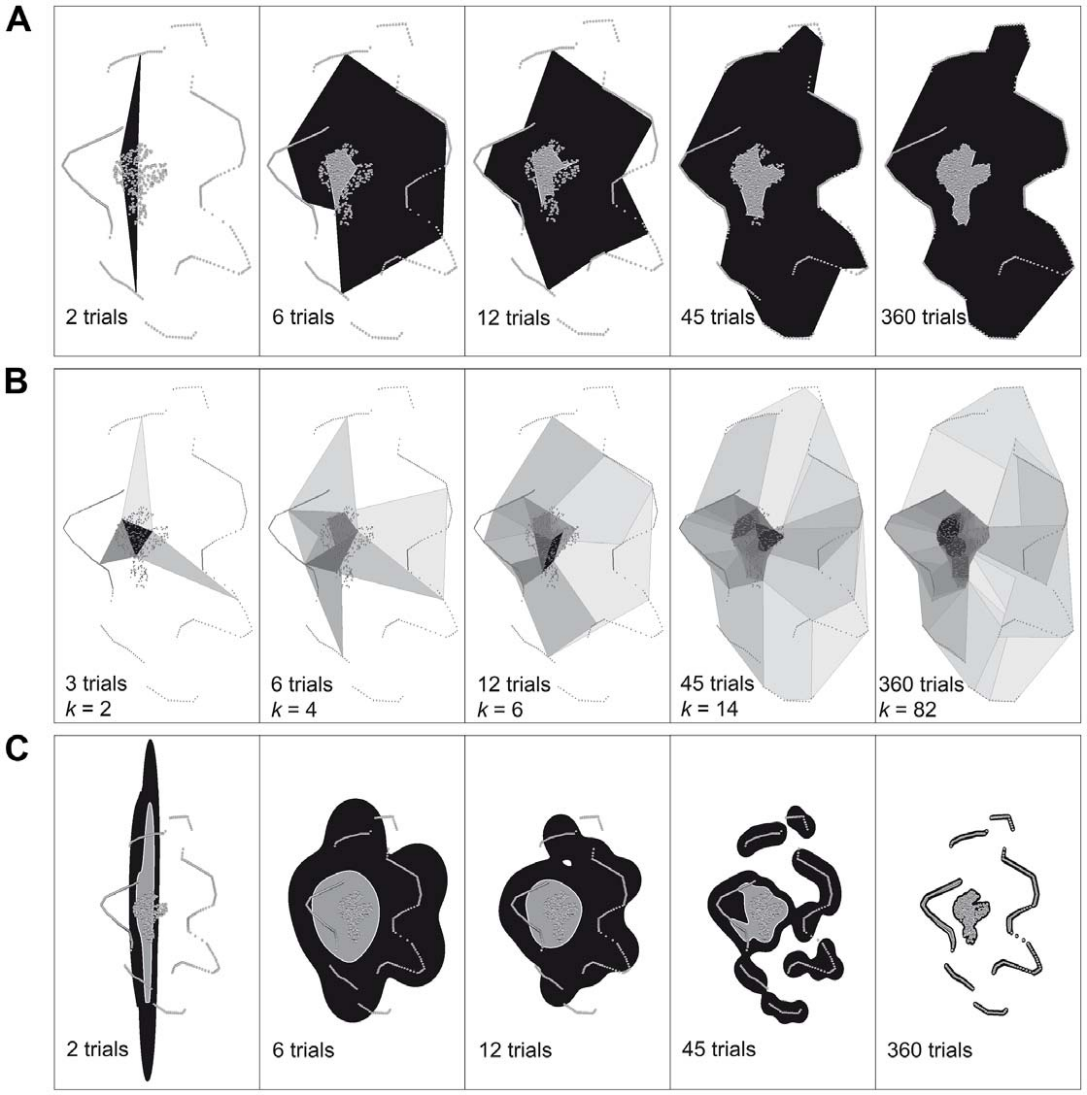
Examples for area estimations. Area estimators of the irregular territory with increasing numbers of equiangular trials, based on the equiangular subsets that include the trial towards direction 0°, the complete series can be found in the supporting figures S4, S5, and S6; A) Detailed hull estimator for the central points (grey area) and the outer points (black area), B) k-LoCoH estimator, areas within the 5% isoclines in incremental shades of grey, C) HRT-LSCV estimator, 95% (black area) and 50%-isoclines (grey area)

Our findings and recommendations concerning the use and performance of area and utilization distribution estimators with point patterns from intrusion experiments depend on the intended use of the estimates. The criteria we used for ‘good’ estimators were asymptotic behaviour (towards ‘true’ absolute territory size) and stable rank order, which also mean that a probably existing bias in absolute territory size acts equally on all individuals under study. The best performing estimator in these terms was the detailed hull estimator (Figs. 5B, 10A), which, on the other hand, faces two caveats. Unlike other detailed descriptors of point patterns, and due to the manufacturer’s copyright policy, DH as implemented in ‘XToolsPro’ [55] is not ‘open source’, although some information regarding the underlying algorithm [56] was disclosed ([65] and personal communication with Data East). Nevertheless we decided to use this estimator in our current study, as it is the only implementation of a detailed polygon descriptor that is readily available to field biologists. As other studies point out, it is likely that other, similar descriptors [40], [64] will perform equally well or better [65]. We urge developers to make their methods and algorithms openly available and implement them for use in widespread software environments. The second drawback of DH, as with any simple point-connecting method, is the lack of an estimation of the internal distribution of space use. In the current context of intrusion trials this may be remedied by the separate calculation of initial (‘calling’) and attracted (‘playback’) territories, which results in the separate delimitation of core and peripheral areas. However the explicit construction of internal utilization distributions remains desirable.

Among all estimators with internal utilization distributions, the non-parametric LoCoH estimator with MSHC adjustment of the tuning parameter *k* (Figs. 5C, 10B) produced the most concise results in terms of rank order and absolute territory size. However, regarding absolute territory size, the LoCoH estimator performed more similar to the MCP than to the DH estimator (Table 1). Both methods overestimated the extension of concave shapes with according fluctuations in rank order stability (Fig. 8). All parametric kernel estimators turned out to be unsuitable for the purpose of area estimation based on point patterns from intrusion experiments as all of them showed erratic fluctuations with increasing sample size (Fig. S1). We attribute this observation to a failure of the various algorithms to determine a sensible smoothing parameter *h* for the kernels. While all of these methods perform reasonably well with point patterns as they typically occur in home range studies (bounded, random patterns with some clumping), the bimodal clumping of points in the central area and along the edge of a territory, as it results from intrusion experiments, is likely to produce estimates of the parameter that are nonsensical, at least for the intended purpose (Figs. 10C, S6). We also noted considerable discordances between different parametric kernel estimator plug-ins that pretended to employ the same algorithms for the calculation of the parameter *h* and for kernel calculation (AM-LSCV vs. ABODE-LSCV vs. HRT-LSCV(f); ABODE-HREF vs. HRT-HREF(f); Fig. S1). This observation was made already for home range studies [70]–[72] and also showed in our analyses of all points as well as of the restricted subset of central points only.

### Frog data

Males of *Allobates femoralis* defend territories that are much larger than the area that comprises the calling locations, consistent with [73]. The analysis of playback trials of 15 individuals with the DH estimator revealed that the extension of the defended area, the ‘playback territory’, does not correlate with the area occupied during calling, the ‘calling territory’. This is of special interest in the context of previous studies where a correlation between the size of the calling territory and reproductive success in this species was initially found [46] but later disputed [11]. In both studies, territory size was determined through mapping of calling positions over several months and the estimation by the modified-minimum-area method [74] to eliminate outliers. In the present study we intentionally did not evaluate the frog’s positions by the MMA method as our observation periods were considerably shorter than those of previous studies, which would have rendered direct comparisons meaningless. Our present findings call for further studies of the effects of territory size on reproductive success in *A. femoralis* and other Dendrobatoids with a distinction between calling and defended territories, and an investigation into their differential roles in the reproductive behaviour of the species.

The comparison of the total extension of DH, LoCoH and STC estimators showed that all three methods produce essentially analogue rank orders (Fig. S3). Most differences originated from the variation in territory shape that resulted from the sensibility of the STC method to trials where tested males showed no reaction in a certain direction. The apparent lack of playback territory overlap (Fig. 4) indicates that this non-responsiveness in certain directions can be interpreted as an exclusion mechanism towards neighbouring territorial males. For only two individuals we could find a spatial overlap of playback territories, however the underlying playback trials were temporally separated by twelve days, which corroborates the notion of exclusive occupancy of territories with dynamic fluctuations over time.

In terms of extension of the isoclines our newly developed STC method yielded highly concordant results with the LoCoH estimator over the whole range of %-isoclines. However, due to the effects described above, there was considerable variation in the shape of %-isoclines, especially at the centre of the territories. For the LoCoH and for the STC estimator, area equality with the DH of the calling territories was reached on average at the 50% and 40%-isoclines, respectively, however complete overlap was reached only as late as at the 80% and 90%-isoclines, respectively. Despite these promising results, there aresome limitations to the STC approach. Clearly it would have been desirable to apply the ‘rubbersheet’ spatial adjustment directly to the underlying probability distributions instead of the resulting isoclines. However, when applied to the raster data of the distributions, the corresponding spline transformation for georeferencing (cf. ArcGIS manual [69] did produce severe artefacts far outside the DH playback territories, rendering the subsequent construction of probability isoclines meaningless. Thus the indirect approach to stretch and jolt isoclines of previous kernel calculations currently is the only practical way to apply the STC method. Given the highly promising results from comparisons with other estimators, we urge for the elaboration and further development of this approach and its implementation in widespread software environments.

Our findings show that an active approach to assess territoriality and territory size can be more appropriate than traditional observational techniques, especially in the analysis of central-place territories. The fact that the extension of observed and defended territories does not correlate in *A. femoralis* (and presumably in other species as well) calls for further research on territorial behaviour, appropriate estimators of territory size, and the influence of territorial behaviour on fitness and reproductive success.

## Supporting Information

Figure S1 **Average values of the different area estimators for equiangular trial-subsets in virtual territories.** A) ellipse, B) star, C) triangle, D) angle, E) circle, F) irregular; horizontal grey line indicates ‘true’ absolute territory size; area in arbitrary units.
(TIF)

Figure S2 **Average values of the different area estimators for point of the central area.** A) ellipse, B) star, C) triangle, D) angle, E) circle, F) irregular; area is given in arbitrary units.
(TIF)

Figure S3 **Rank order of 15 territories as evaluated by the LoCoH, DH, and STC estimator.**
(TIF)

Figure S4 **Example for the detailed hull estimator.** Detailed hull of the Central points (grey area) and outer points (black area) of the irregular virtual territory with increasing numbers of equiangular trials, based on all equiangular subsets that include the trials towards direction 0°.
(TIF)

Figure S5 **Example of the LoCoH estimator.** LoCoH of the irregular virtual territory with increasing numbers of equiangular trials, based on all equiangular subsets that include the trials towards direction 0°; areas of stepwise increasing 5% isoclines in incremental shades of grey.
(TIF)

Figure S6 **Example for the HRT-LSCV estimator.** 95% (black area) and 50%-isoclines (grey area) of the HRT-LSCV estimator of the irregular virtual territory with increasing numbers of equiangular trials, based on all equiangular subsets that include the trials towards direction 0°.
(TIF)
