# Supplementary figures and images for: Intrusion Experiments to Measure Territory Size: Development of the Method, Tests through Simulations, and Application in the Frog Allobates femoralis
Source: PLoS One. 2011 Oct 14;6(10):e25844. doi: 10.1371/journal.pone.0025844 (PMC3194823; doi:10.1371/journal.pone.0025844)

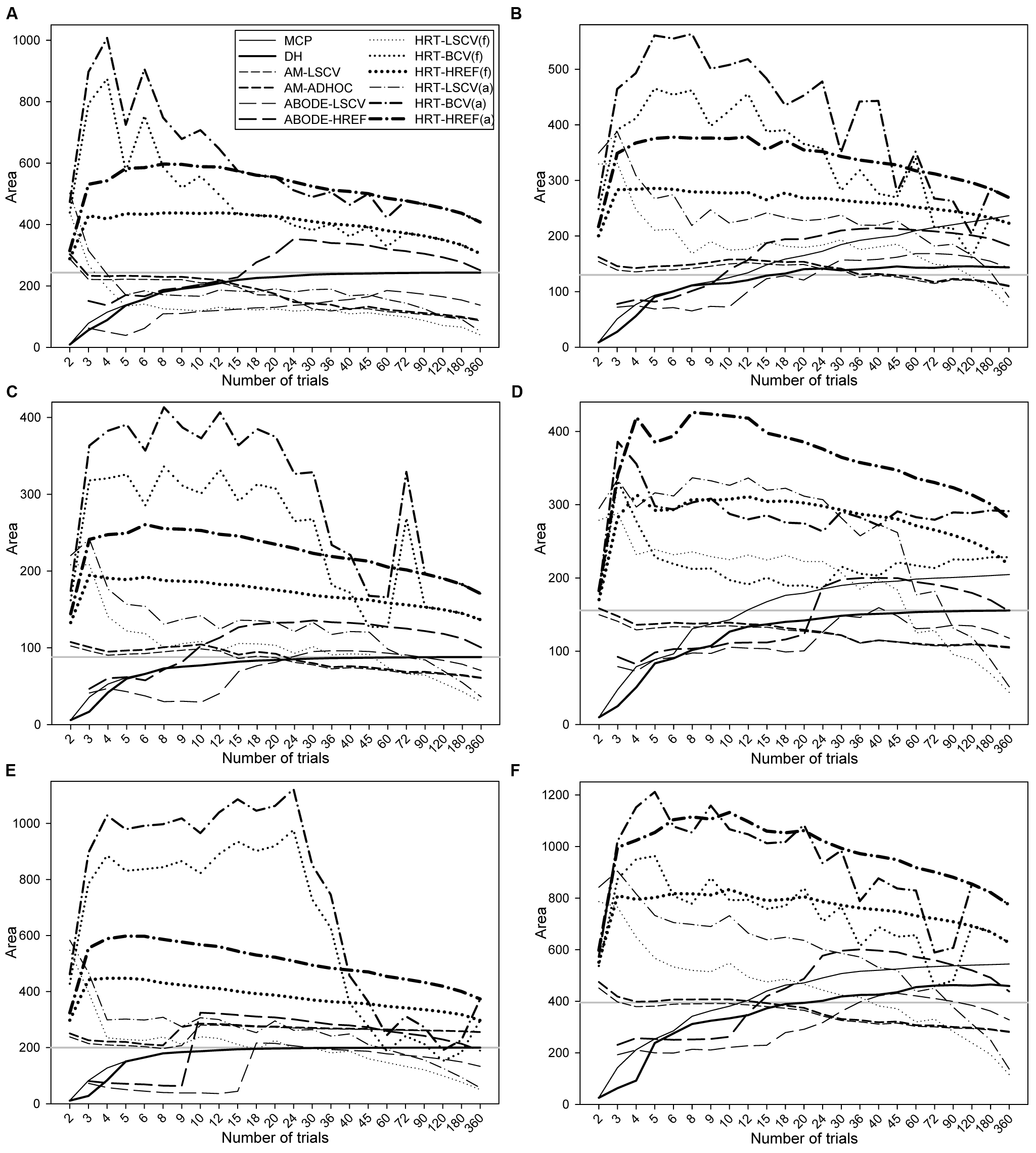

Supplement: Figure S1 — Average values of the different area estimators for equiangular trial-subsets in virtual territories. A) ellipse, B) star, C) triangle, D) angle, E) circle, F) irregular; horizontal grey line indicates ‘true’ absolute territory size; area in arbitrary units. (TIF) [file pone.0025844.s001.tif]

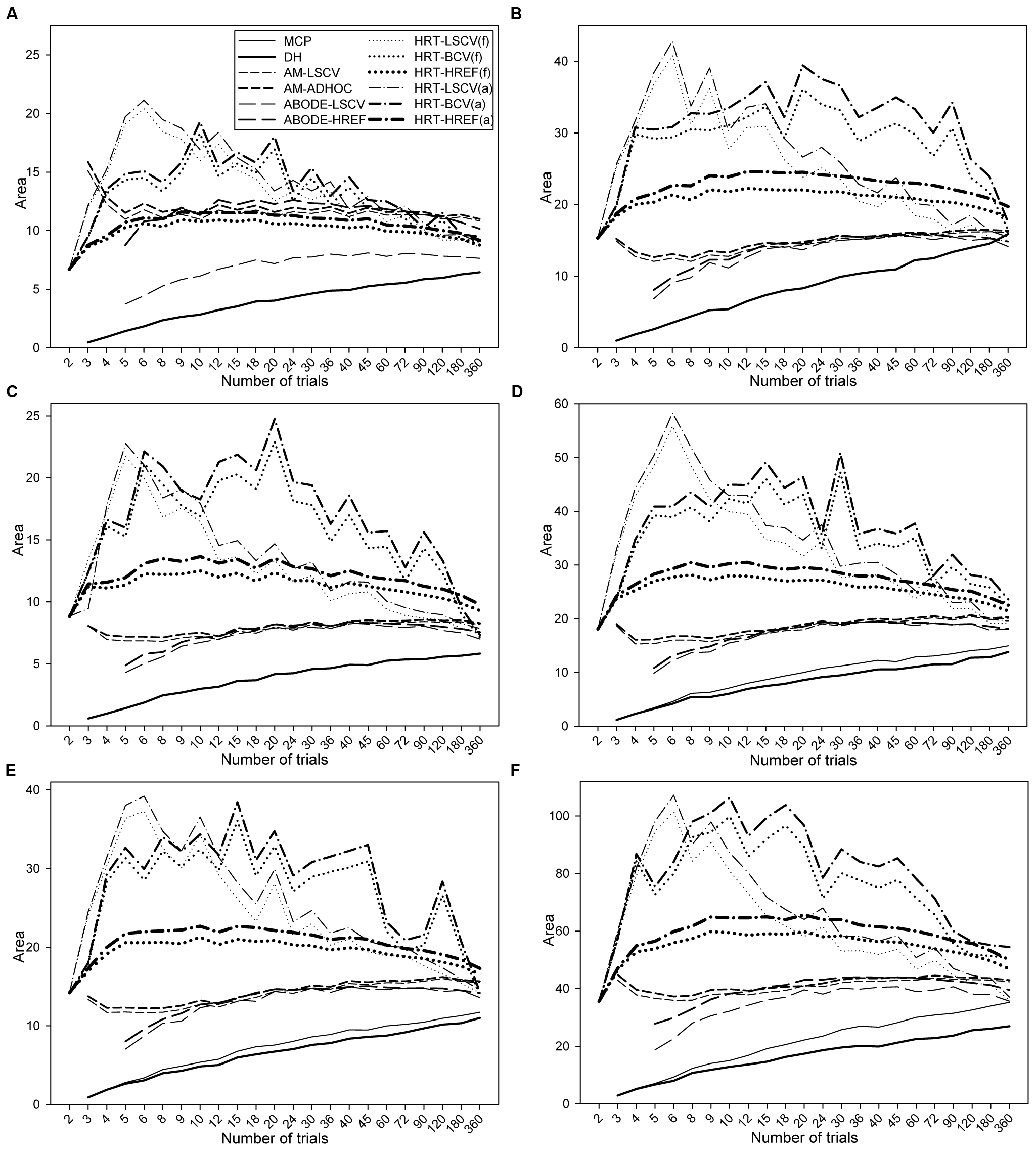

Supplement: Figure S2 — Average values of the different area estimators for point of the central area. A) ellipse, B) star, C) triangle, D) angle, E) circle, F) irregular; area is given in arbitrary units. (TIF) [file pone.0025844.s002.tif]

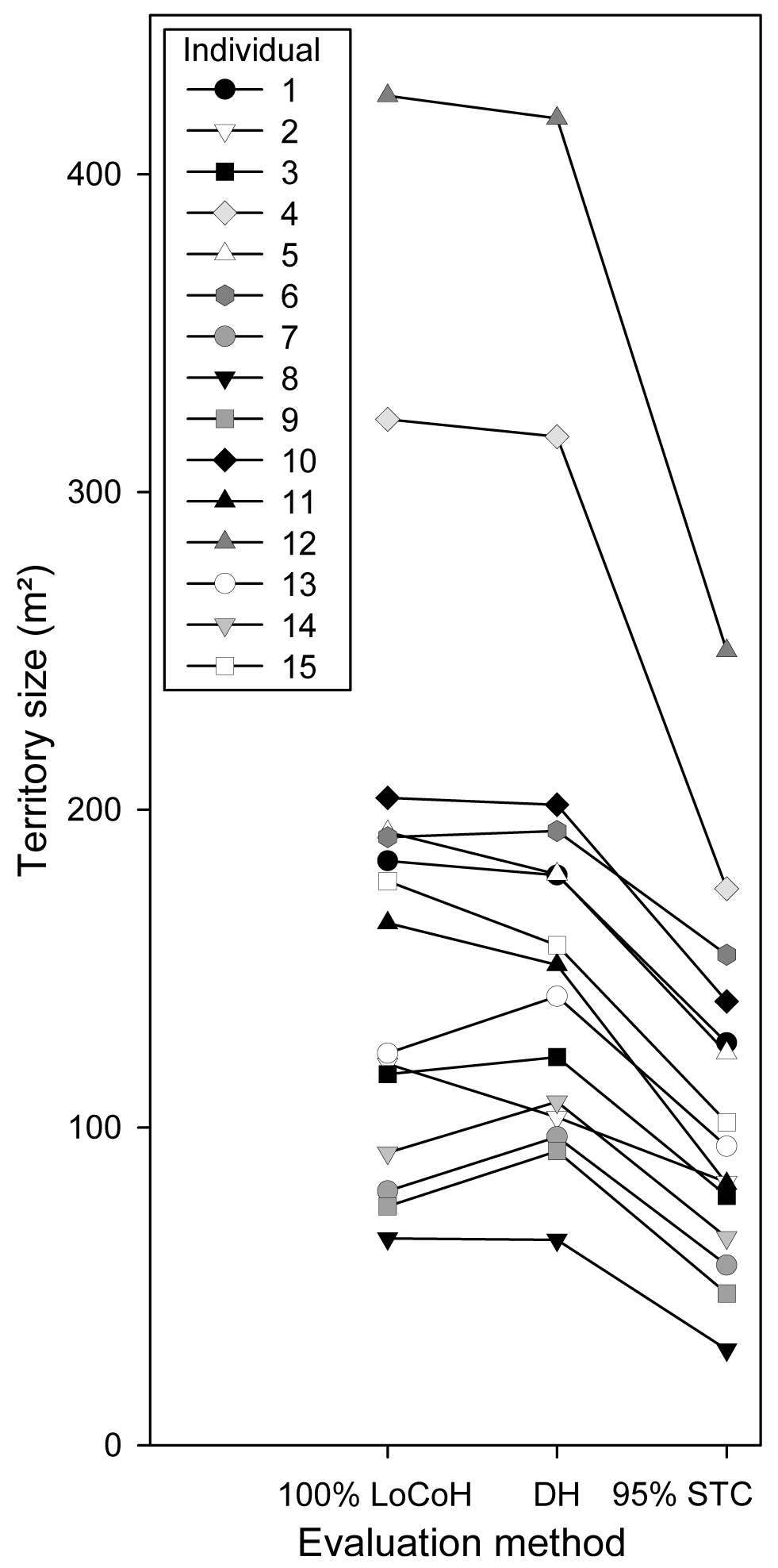

Supplement: Figure S3 — Rank order of 15 territories as evaluated by the LoCoH, DH, and STC estimator. (TIF) [file pone.0025844.s003.tif]

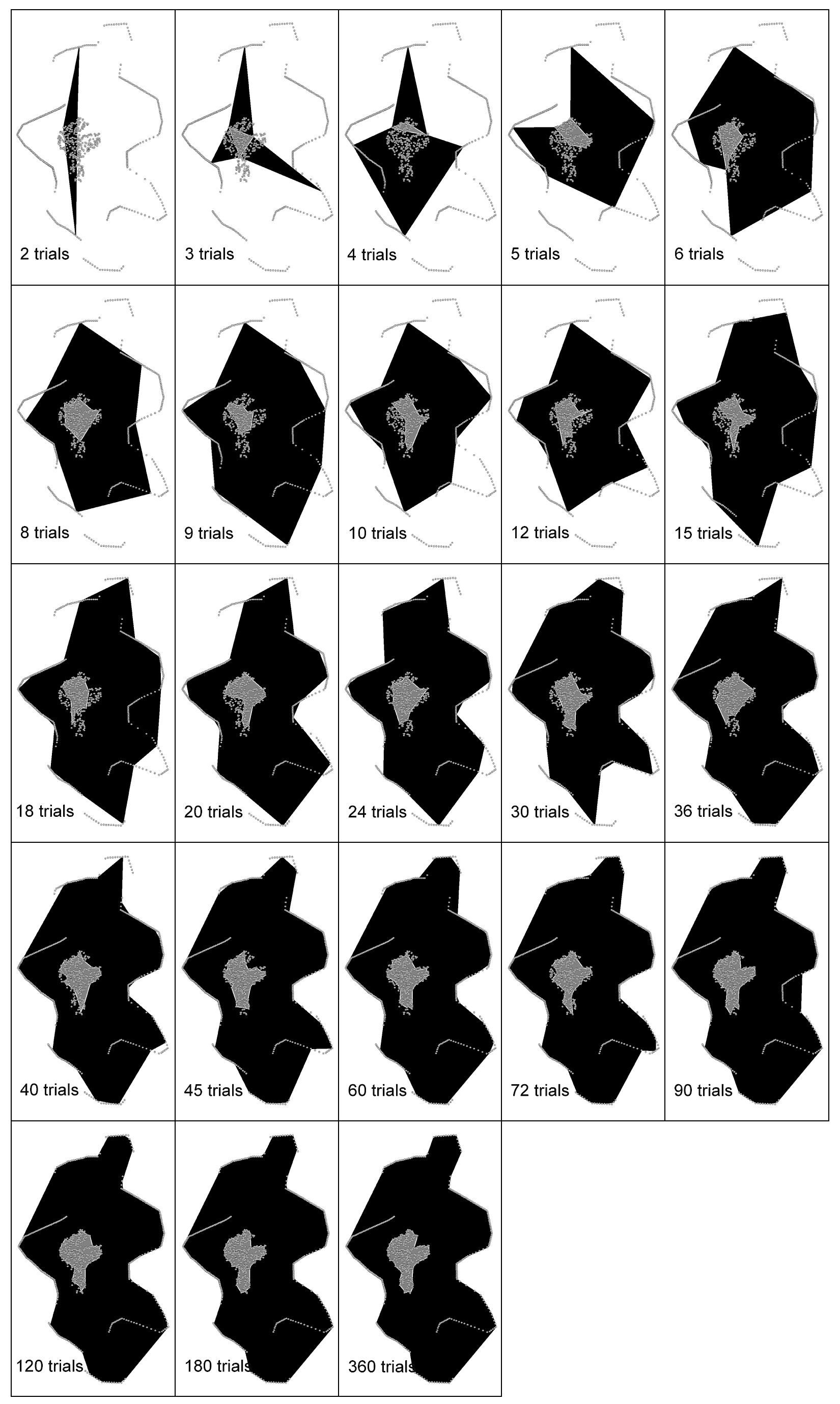

Supplement: Figure S4 — Example for the detailed hull estimator. Detailed hull of the Central points (grey area) and outer points (black area) of the irregular virtual territory with increasing numbers of equiangular trials, based on all equiangular subsets that include the trials towards direction 0°. (TIF) [file pone.0025844.s004.tif]

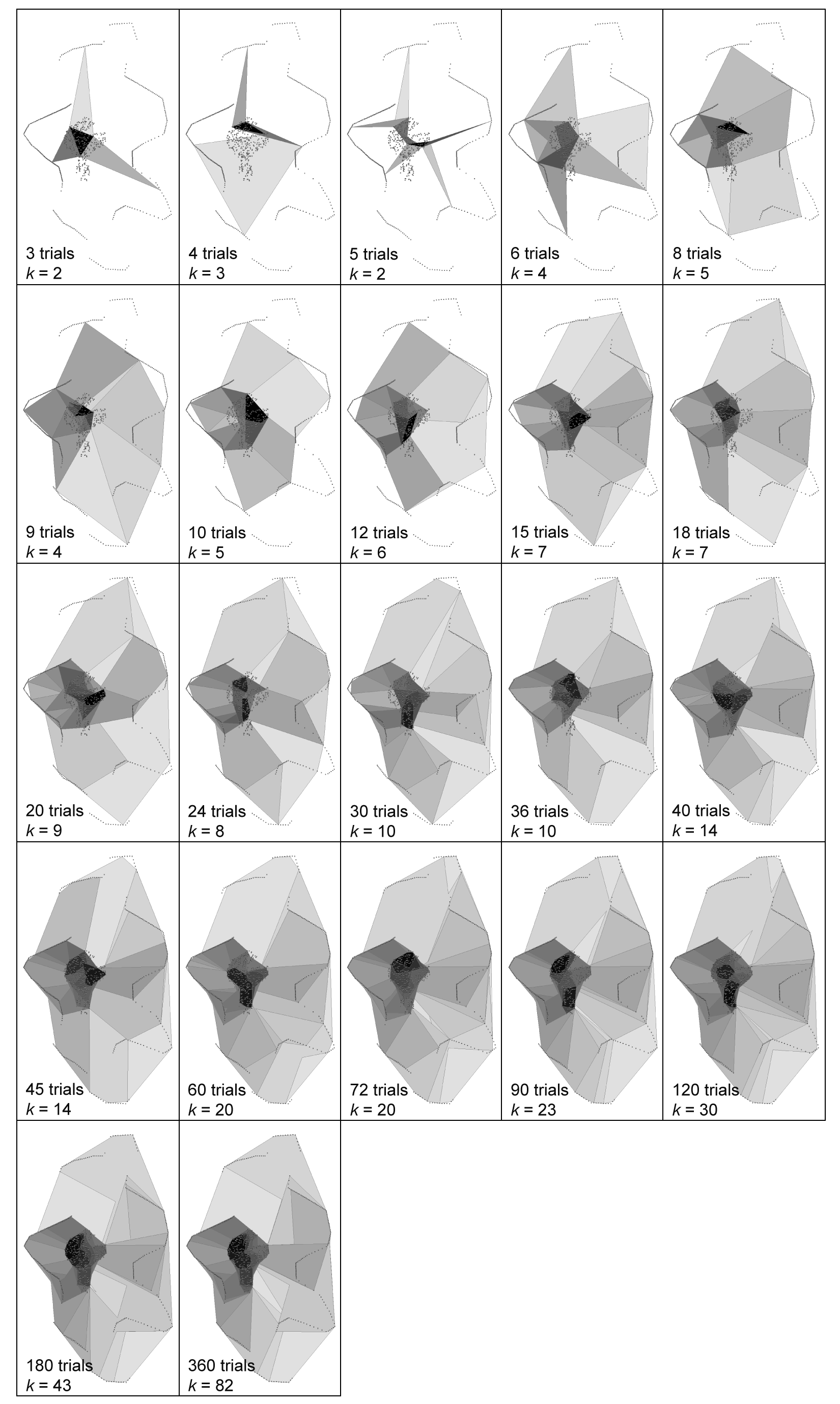

Supplement: Figure S5 — Example of the LoCoH estimator. LoCoH of the irregular virtual territory with increasing numbers of equiangular trials, based on all equiangular subsets that include the trials towards direction 0°; areas of stepwise increasing 5% isoclines in incremental shades of grey. (TIF) [file pone.0025844.s005.tif]

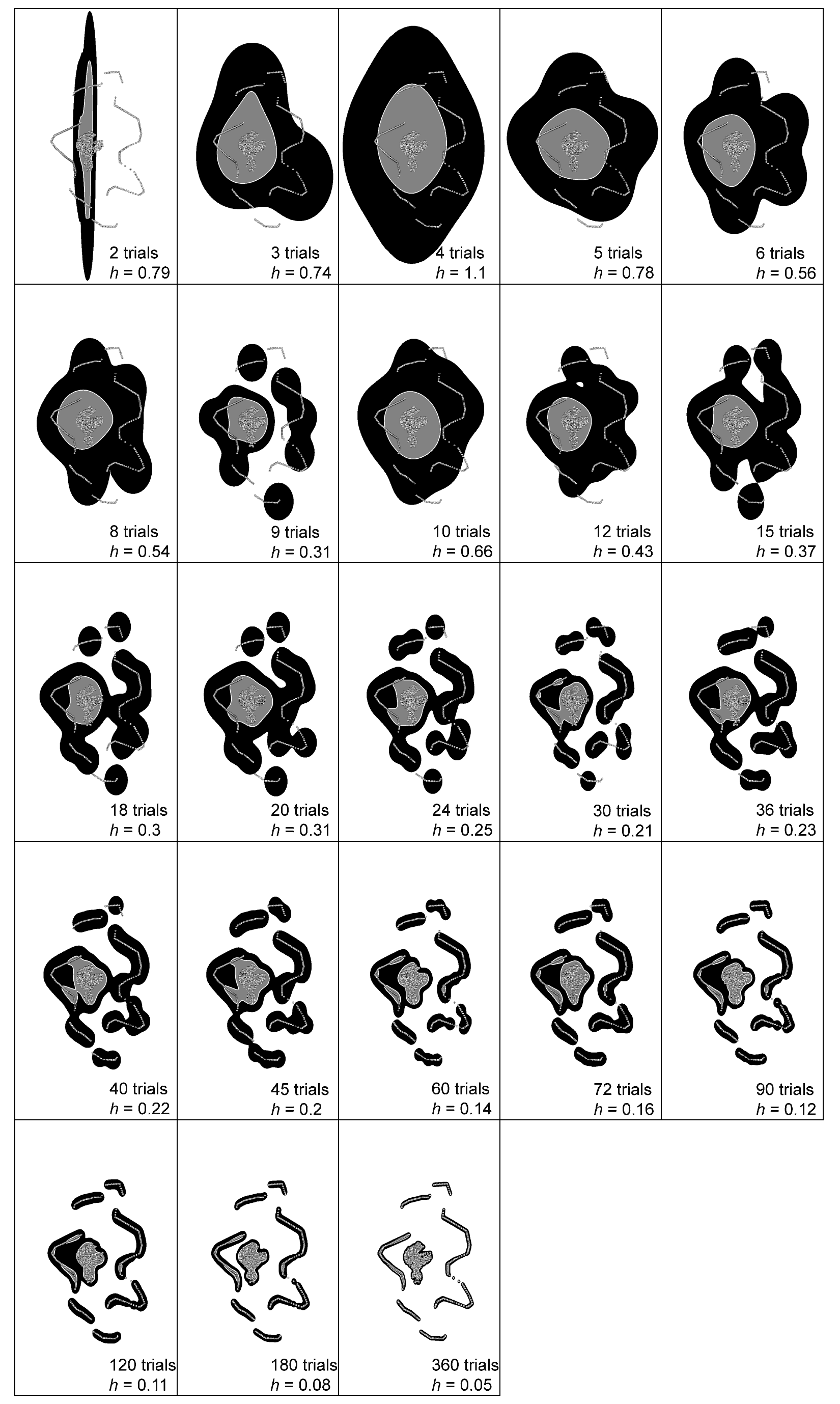

Supplement: Figure S6 — Example for the HRT-LSCV estimator. 95% (black area) and 50%-isoclines (grey area) of the HRT-LSCV estimator of the irregular virtual territory with increasing numbers of equiangular trials, based on all equiangular subsets that include the trials towards direction 0°. (TIF) [file pone.0025844.s006.tif]
